# ﻿*Lagerstroemiastenophylla* (Lythraceae), a new species from China

**DOI:** 10.3897/phytokeys.234.111861

**Published:** 2023-10-13

**Authors:** Bao-Huan Wu, Xing Hu, Wen-Hui Tu, Wei Wang, Se-Ping Dai

**Affiliations:** 1 Guangzhou Institute of Forestry and Landscape Architecture, Guangzhou 510405, China Guangzhou Institute of Forestry and Landscape Architecture Guangzhou China; 2 Guangzhou Horticultural Plant Germplasm Resource Nursery, Guangzhou 510405, China Guangzhou Horticultural Plant Germplasm Resource Nursery Guangzhou China; 3 Guangzhou Collaborative Innovation Center on Science-Tech of Ecology and Landscape, Guangzhou 510405, China Guangzhou Collaborative Innovation Center on Science-Tech of Ecology and Landscape Guangzhou China

**Keywords:** China, crape myrtle, Flora, new species

## Abstract

*Lagerstroemiastenophylla*, a new species from southeastern Shaanxi Province and northwestern Hubei Province of China is described. Morphologically, *L.stenophylla* resembles *L.subcostata*, but it differs in having 4-angular, subalate branchlets, elliptic-lanceolate, or narrowly elliptic leaves, and relatively larger flowers.

## ﻿Introduction

*Lagerstroemia* L. (Lythraceae) is a genus of trees or shrubs with simple leaves, terminal panicles of showy flowers, and woody capsule fruits (Qin and Graham 2007, De Wilde and Duyfjes 2014). *Lagerstroemia* is one of the most popular ornamental flowering trees in China, producing showy flowers in summer. One such is the *Lagerstroemiaindica* L., also known as Bairihong (百日红) in Chinese, which can bloom for up to 100 days, and is widely cultivated in China (Wang et al. 2022). In taxonomy, the monograph of this genus was completed by Koehne (1903) and fully revised by Furtado and Srisuko (1969), accepting 53 species. Thereafter, regional taxonomic revisions (Lee and Lau 1983; Hewson 1990; Qin and Graham 2007; De Wilde and Duyfjes 2013, 2014, 2016) and some sporadic taxonomic works (Zhou et al. 2004; Gu et al. 2012; Gu et al. 2015; Deepu and Pandurangan 2017; Pham et al. 2017; De Wilde and Duyfjes 2019) were published successively. In these works, many species were reduced to synonyms, and many new species were described. According to our statistics, the genus *Lagerstroemia* currently comprises about 51 species (excluding subspecies and varieties).

In China, there are 15 *Lagerstroemia* species that have been documented in “Flora of China” (Qin and Graham 2007). *Lagerstroemiaparviflora* Roxb. was recorded in Yingjiang County of southern Yunnan Province (Yuan 1983), and *L.minuticarpa* Debberm. ex P.C.Kanjilal was recorded in Motuo County of Tibet Autonomous Region (Tang 1986), but neither of them was included in “Flora of China”. Recently, two new species of China were discovered, including *L.densa* C.H.Gu & D.D.Ma (Gu et al. 2015) from Guangxi Zhuang Autonomous Region and *L.menglaensis* C.H.Gu, M.C.Ji & D.D.Ma (Gu et al. 2012) from Yunnan Province.

During our examination of *Lagerstroemia* specimens, some collections from south-eastern Shaanxi Province of China, such as *Z.B. Wang 16543* from Shanyang County and *B.Z. Guo 2225* from Xunyang County, were found likely misidentified as *L.subcostata* Koehne. While morphologically similar, these specimens have conspicuously smaller, narrower leaves. A more extensive examination of specimens and literature survey (Koehne 1903; Furtado and Srisuko 1969; Ho 1974; Lee and Lau 1983; Fu 2002; Zhou et al. 2004; Qin and Graham 2007; Gu et al. 2012; De Wilde and Duyfjes 2013, 2014, 2016; Li and Li 2013; Gu et al. 2015; Liu et al. 2022) were therefore conducted. Field investigation was also conducted, and more specimens were collected. The result of these studies confirmed a new *Lagerstroemia* species from Shaanxi and Hubei Provinces of China, which is described below, bringing the total numbers of species of *Lagerstroemia* to 52.

## ﻿Material and methods

Morphological descriptions were based on observations of the living plants in the field and dried specimens in herbaria. Measurements were conducted manually with rulers or using Digimizer version 4.6.0 (MedCalc Software, Mariakerke, Belgium), and a total of 53 collections were measured. The voucher specimens were deposited in Guangzhou Institute of Forestry and Landscape Architecture, and the herbarium of South China Botanical Garden (**IBSC**).

## ﻿Taxonomic treatment

### ﻿ Lagerstroemia stenophylla

Taxon classificationPlantaeMyrtalesLythraceae

B.H.Wu, X.Hu & S.P.Dai sp. nov.

60D94E7A-46CA-5346-A7D8-6EFCA3FF7B44

urn:lsid:ipni.org:names:77328633-1

Fig. 1

#### Type.

China. Shannxi: Shangluo, Jinsixia Town, Xinglong Country, in ravine, on rocky slopes. 33°26'36.14"N, 110°32'51.13"E, 387 m a.s.l., 6 June 2023 (fl.), *B.H. Wu & W.H. Tu Lg202334* (Holotype: IBSC!; isotypes: Herbarium of Guangzhou Institute of Forestry and Landscape Architecture!).

#### Diagnosis.

*Lagerstroemiastenophylla* is morphologically similar to *L.subcostata* Koehne, but distinguished by its branchlets 4-angular, sometimes subalate, leaves elliptic-lanceolate, narrowly elliptic, leaf apex acute, leaf base cuneate, flowers 2.5–3 cm in diameter.

#### Description.

Shrubs or small trees ca. 0.5–3 m tall. Bark reddish brown, longitudinally fissured and slightly peeling to reveal the inner bark when mature; branchlets scabridulous to glabrous, 4-angular, sometimes subalate. Leaves mostly alternate, or subopposite; petiole 1–3 (4) mm long, densely scabridulous to glabrous; leaf blade herbaceous, margin entire, lanceolate to elliptic-lanceolate, rarely ovate or obovate-oblanceolate, 2–6 (7) × 0.7–2 (2.3) cm, base cuneate, apex acute, sometimes obtuse, rarely apiculate, abaxially pale green, scabridulous (especially along midrib and later veins) to glabrous, adaxially green, sparsely scabridulous to glabrous, lateral veins 4–7 pairs. Inflorescences paniculate, terminal or axillary; panicles (2) 4–7 cm long, densely scabridulous. Flowers sessile or subsessile; flower buds turbinate to subglobose (excluding pseudopedicels). Calyx tubes (excluding pseudopedicels) cup-shaped, 3.3–4.2 mm long, outside densely scabridulous to glabrous, with 10–12 distinctly ribs or dark veins (sometimes not obvious), inside glabrous, with glabrous annulus (sometimes absent) in the throat, lobes 6, sometimes 5, triangular, 1–2.3 mm × 1–2.3 mm, erect, epicalyx absent, pseudopedicel 2–9 mm long; petals 6, crumpled, oblong, suborbicular or ovate, base cuneate to broadly cuneate, rounded, or sometimes subcordate, apex obtuse or rounded, 10–18 mm with claws 3–8 mm long; stamens 20–28, dimorphic, with 6 stamens longer (ca. 15 mm long), thicker and red-brown in color, the remaining stamens are shorter (ca.7 mm long), thinner and white in color, filaments glabrous; ovary glabrous, styles 10–14 mm long, glabrous, stigmas small. Capsules globose to oblong, 6–8 mm long, 4.5–6 mm in diameter, loculicidally dehiscent, 4–7-valved. Seeds ca. 6mm long including wing.

**Figure 1.**
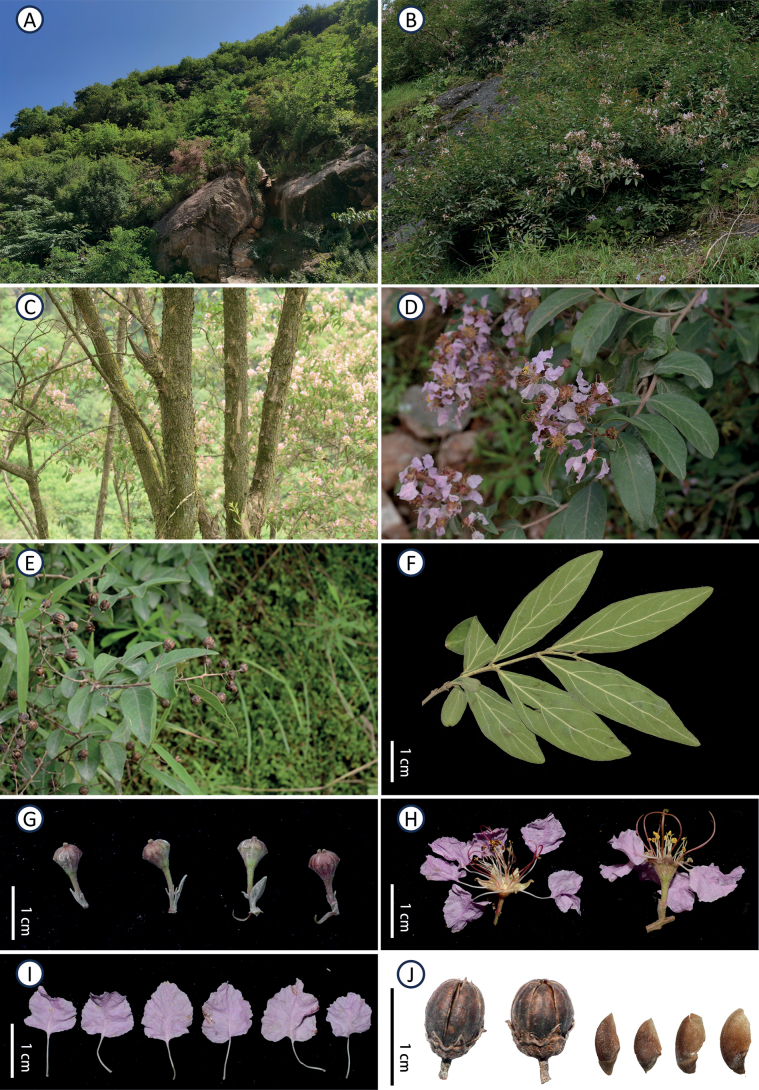
Plate of *Lagerstroemiastenophylla***A** habitat **B** flowering individuals **C** stems **D** flowering branch **E** fruiting branch **F** leaves **G** flower buds **H** flowers **I** petals **J** capsules and seeds.

#### Phenology.

Flowering from May to June, and fruiting after July and fruits persist through winter.

#### Distribution and habitat.

**Figure 2.**
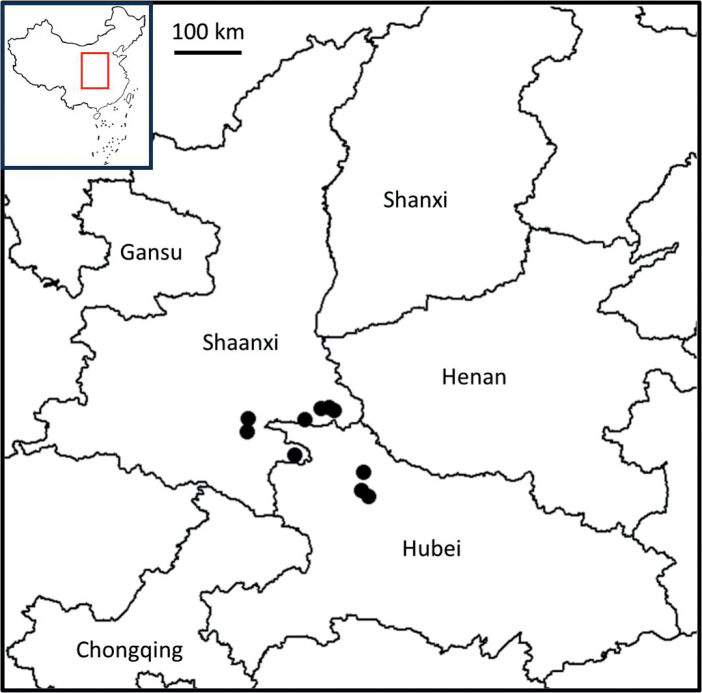
Distribution of *Lagerstroemiastenophylla*.

*Lagerstroemiastenophylla* is hitherto known from Baihe County, Danfeng County, Shanyang County, Shangnan County, Xunyang County, Zhashui County, Zhen’an County of south-eastern Shaanxi Province and Baokang County, Fang County and Shiyan City of north-western Hubei Province (Fig. 2). It grows on rocky slopes in ravine, at 290–770 m elevation.

#### Etymology.

The epithet of the new species refers to its narrow leaf blade.

#### Vernacular name.

The Chinese name of the new species is here given as 狭叶紫薇 (xiá yè zǐ wēi).

#### Note.

*Lagerstroemiastenophylla* is formerly misidentified as *L.subcostata* (Li and Li 2013). Morphologically, *L.stenophylla* and *L.subcostata* share many similar characters, including calyx tubes cup-shaped, with ca. 12 ribs or darkened veins, epicalyx absent, and sepals adaxially glabrous, stamens less than 30, leading to misidentify *L.stenophylla* as *L.subcostata*, using the Keys of “Flora Reipublicae Popularis Sinicae” (Lee and Lau 1983) and “Flora of China” (Qin and Graham 2007). However, *L.stenophylla* is a shrub or small tree with conspicuously smaller, narrower leaves and larger flowers, differing distinctly from *L.subcostata*. Detailed morphological comparisons among *L.stenophylla* and its relatives are presented in Table 1.

**Table 1.** Morphological Comparison of *Lagerstroemiastenophylla* and its relatives.

Characters	*L.stenophylla*	*L.subcostata*	*L.indica*	*L.excelsa*
Branchlets	4-angular, sometimes subalate	terete to slightly 4-angular	4-angled or subalate	terete
Leaf shape	elliptic-lanceolate, narrowly elliptic, rarely ovate, obovate-oblanceolate	oblong, ovate-lanceolate, elliptic, obovate-elliptic, or infrequently obovate	elliptic, oblong, obovate, or suborbicular	elliptic to broadly elliptic
Leaf size	2–6 (7) × 0.7–2 (2.3) cm	2–9 (11) × 1–5 cm	2.5–7(10) × 1.5–4 cm	7–13 × 3.5–5 cm
Leaf apex	acute, sometimes obtuse, rarely apiculate	acuminate	acute, obtuse with small mucro, or retuse	narrowly to broadly acuminate
Leaf base	cuneate	broadly cuneate to subrounded	broadly cuneate to rounded	acute
Lateral veins	4–7 pairs	3–10 pairs	3–7 pairs	7–9 pairs
Flower	2.5–3 cm in diameter	less than 1 cm in diameter	3–4 cm in diameter	ca. 0.5 cm in diameter
Calyx-tube	cup-shaped, with 10–12 distinctly ribs or dark veins, sometimes not obvious	cup-shaped, with 10–12 dark veins or faint ribs	campanulate, obscurely to decidedly 6-ribbed	tubular, with 12 dark veins or ribs
Annulus	present, sometime absent	thin or apparently absent	present	absent
Petal	10–18 mm long including claw	2–6 mm long including claw	12–20 mm long including claw	3–3.5 mm long including claw
Stamens	20–28	15–30	36–42	(5)6–12
Seed	ca. 6 mm long including wing	ca. 4 mm long including wing	ca. 8 mm long including wing	3.5–4.8 mm long including wing
Phenology	flowering from May to June, fruiting after July	flowering from June to August, fruiting from July to October	flowering from June to September, fruiting from September to November	flowering in April, fruiting in July

#### Additional specimen examined.

**China. Shaanxi Province**: Baihe County, Maoping, 29 September 1969, *Vegetation Team 541* (WUK); *ibid.*, 8 June 2023, *B.H. Wu and W.H. Tu Lg202378*, *Lg202379*, *Lg202380*, *Lg202381*; Danfeng County, Tumen Town, 6 June 2023, *B.H. Wu and W.H. Tu Lg202329*, *Lg202331*; Danfeng County, Zhulinguan Town, Baijiawan, 6 June 2023, *B.H. Wu and W.H. Tu Lg202332*; Shanyang County, Zhaochuan, 1 July 1960, *Huashan Team 0206* (IBSC); Shanyang County, Manchuanguan, 17 September 1952, *Z.B. Wang 16543* (KUN, PE, WUK); *ibid.*, 1 May 1964, *J.X. Yang 2456* (WUK); *ibid.*, 19 May 2011, *S.F. Li et al. 15016* (XBGH); *ibid.*, 5 June 2023, *B.H. Wu and W.H. Tu Lg202312*, *Lg202313*, *Lg202314*, *Lg202315*, *Lg202316*, *Lg202317*, *Lg202318*, *Lg202319*; Shangnan County, Jinsixia, 6 June 2023, *B.H. Wu and W.H. Tu Lg202335*, *Lg202336*, *Lg202337*, *Lg202338*; Xunyang County, on the road from Zhaojiawan to Liangheguan, 7 October 1952, *B.Z. Guo 2225* (WUK); Xunyang County, precise locality unknown, 1959, *Xida 019* (WUK); Xunyang County, on the road from Zhangping to Xunyang, 19 August 1959, *P.Y. Li 8962* (WUK); Xunyang County, Guojiacao, 7 May 2012, *S.F. Li et al. 16523* (XBGH); Xunyang County, Liangheguan, 9 June 2023, *B.H. Wu and W.H. Tu Lg202384*, *Lg202385*, *Lg202386*, *Lg202387*, *Lg202388*; Zhashui County, Shiweng, 8 June 2008, *S.F. Li et al. 10463*; Zhen’an County, Lengshuihe, 9 June 2023, *B.H. Wu and W.H. Tu Lg202389*, *Lg202390*, *Lg202392*, *Lg202393*. **Hubei Province**: Baokang County, Siping, 21 April 1986, *84Linxue 86-1058* (CCAU); *ibid.*, 8 June 2023, *B.H. Wu and W.H. Tu Lg202365*, *Lg202366*, *Lg202367*, *Lg202369*, *Lg202372*; Fang County, Wanyuhe, 8 June 2023, *B.H. Wu and W.H. Tu Lg202373*, *Lg202374*, *Lg202375*; Shiyan City, Wudangshan, 7 June 2023, *B.H. Wu and W.H. Tu Lg202343*, *Lg202344*, *Lg202345*, *Lg202346*, *Lg202347*, *Lg202348*.

## Supplementary Material

XML Treatment for Lagerstroemia stenophylla

## ﻿Additional information

### Author ORCIDs

Bao-Huan Wu https://orcid.org/0000-0002-0498-0603

### Data availability

All of the data that support the findings of this study are available in the main text.
